# Effect of Decorin and Aligned Collagen Fibril Topography on TGF-β1 Activation of Corneal Keratocytes

**DOI:** 10.3390/bioengineering12030259

**Published:** 2025-03-05

**Authors:** Nathaniel S. Tjahjono, Divya Subramanian, Tarik Z. Shihabeddin, Hudson D. Hicks, Victor D. Varner, W. Matthew Petroll, David W. Schmidtke

**Affiliations:** 1 Department of Bioengineering, University of Texas at Dallas, Richardson, TX 75080, USA; nathaniel.tjahjono@utdallas.edu (N.S.T.); divya.subramanian@utdallas.edu (D.S.); tarik.shihabeddin@utdallas.edu (T.Z.S.); hudson.hicks@utdallas.edu (H.D.H.); vdv@utdallas.edu (V.D.V.); 2 Department of Biomedical Engineering, University of Texas Southwestern Medical Center, Dallas, TX 75390, USA; matthew.petroll@utsouthwestern.edu; 3 Department of Ophthalmology, University of Texas Southwestern Medical Center, Dallas, TX 75090, USA

**Keywords:** decorin, aligned collagen fibrils, corneal keratocytes, transforming growth factor-beta, alpha-smooth muscle actin

## Abstract

During corneal wound healing, transforming growth factor-beta 1 (TGF-β1) causes differentiation of quiescent keratocytes into myofibroblasts. Decorin has been investigated as a promising anti-fibrotic therapeutic for corneal healing due to its interaction with TGF-β1, collagen, and cell surface receptors. In this study, a novel microfluidic method for coating aligned collagen fibrils with decorin was developed to mimic the presence of decorin within the corneal stroma. Decorin was found to bind selectively to collagen and remained bound for at least five days. To investigate the effects of decorin coatings on keratocyte activation, primary rabbit keratocytes were cultured in the presence of TGF-β1 for 5 days on substrates with or without decorin and stained for α-smooth muscle actin (α-SMA). The expression of α-SMA was reduced by similar amounts on monomeric collagen (40%), random collagen fibrils (32%), and aligned collagen fibrils (32%) coated with decorin as controls. However, α-SMA expression was differentially expressed between the collagen substrates not coated with decorin, with significantly lower expression on uncoated aligned collagen fibrils compared to uncoated collagen monomers. Addition of decorin directly to culture media, had a limited effect on reducing myofibroblast differentiation. Taken together, these results demonstrate the importance of topography and ECM composition on keratocyte activation.

## 1. Introduction

In the corneal stroma, transparency is maintained through an interplay between resident keratocytes and their highly organized extracellular matrix (ECM), consisting primarily of aligned collagen I fibrils arranged in orthogonally stacked layers known as lamellae [1,2]. In addition to fibrillar collagen, the corneal ECM contains a multitude of other proteins, including proteoglycans such as decorin, keratocan, and lumican, which play important roles in corneal function and maintenance [3]. While corneal keratocyte-ECM interactions have been investigated largely within fibrillar collagen matrices [4,5,6,7] or protein-functionalized surfaces [8,9,10,11], less is known about the contributions of individual proteoglycans, such as decorin, to corneal keratocyte differentiation into myofibroblasts.

Decorin is a small leucine-repeat proteoglycan secreted in the corneal stroma [12] with high affinity for collagen I, cell surface receptors, such as epidermal growth factor receptor (EGFR), low-density lipoprotein receptor-related protein one (LRP-1), or platelet-derived growth factor receptor (PDGFR) [13,14,15,16], and growth factors such as transforming growth factor-beta 1 (TGF-β1), platelet-derived growth factor (PDGF), or insulin-like growth factor (IGF) [16,17,18,19]. Because of its versatility in binding partners, decorin has been suggested to play a crucial role in various corneal functions, including regulation of collagen fibril diameter and spacing during corneal development and wound healing [20,21,22]. In wound healing, decorin has been implicated as an inhibitor of fibrosis when delivered either as a wound dressing in a murine model or through targeted gene therapy in a rabbit model [23]. The current hypothesis for the mechanism behind this fibrosis inhibition suggests that the binding of decorin to TGF-β1, which is known to mediate myofibroblast differentiation, prevents TGF-β1 binding to cell surface receptors and thus disrupt its downstream signaling [17]. Further validation and exploration of this hypothesis would benefit from in vitro studies in which specific cues, such as decorin, collagen fibril topography, or growth factor presence, can be isolated and controlled independently to determine their interactions and potential synergistic or antagonistic effects.

While several previous studies have reported the inhibition of TGF-β1 mediated corneal fibrosis by decorin [23,24,25], to our knowledge, there are no studies that evaluate the combination of decorin with the topography of aligned collagen fibrils native to the corneal stroma on TGF-β1 mediated keratocyte activation. This gap in knowledge presents a deficiency in the relevance of current data due to both the importance of ECM organization and alignment in the cornea, and reports that topography can modulate keratocyte activation in response to TGF-β1 [11,26].

In this study, we investigated the effects of decorin incorporation into aligned collagen fibrils on corneal keratocyte response to TGF-β1 and found that inhibition of TGF-β1 mediated differentiation was dependent on collagen topography, decorin concentration, and the method used to incorporate decorin into the collagen fibrils.

## 2. Materials and Methods

### 2.1. Micropatterning of Aligned Collagen Fibrils

The micropatterns of aligned collagen fibrils were deposited as previously described [27,28]. Briefly, polydimethylsiloxane (PDMS) straight microfluidic channels (750 μm wide) were exposed to air plasma for 1 min on high RF at 350 mTorr using a Harrick Plasma Cleaner (Harrick Plasma; Ithaca, NY, USA) and bonded to glass coverslips previously spin-coated with PDMS (1000 RPM for 30 s). The devices were placed on an aluminum hotplate (Torrey Pines Scientific; Carlsbad, CA, USA) set to 40 °C for 30 min in a cold room (4 °C) prior to perfusion to ensure a tight seal and equilibrate device temperature. A collagen solution was then prepared by mixing type I bovine collagen (3.0 mg/mL) (Advanced BioMatrix; Carslbad, CA, USA) with 10× minimal essential media (MEM) (Life Tech; Carlsbad, CA, USA) and 0.1 M NaOH in an 8:1:1 volume ratio. To adjust the final collagen solution to 1.6 mg/mL 1× MEM was added, and additional NaOH was added as needed to reach a final pH of 7.55–7.6. Perfusion of the solution through the microfluidic devices was performed at a shear rate of 200 s^−1^ for 8 min, or until the solution reached the channel outlet, and then at a shear rate of 150 s^−1^ for 30 min. Excess collagen solution was pipetted from the channel outlet and fibril patterns were washed by withdrawing 1× PBS through the channel for 10 min at 40 μL/min. The PDMS channels were removed from PDMS-coated coverslips, and the patterns were dried on a hot plate (37 °C) for 30 min.

### 2.2. Patterning of Random Collagen Fibrils and Collagen Monomer Coated Surfaces

Random collagen fibrils were deposited as previously described [28]. First, PDMS rings were bonded to the PDMS spin-coated glass coverslips and placed on an aluminum hotplate set to 40 °C. Then, 1 mL of the 1.6 mg/mL collagen solution prepared as described above was added to each PDMS ring and left for 5 min on the hotplate. Next, the substrates were placed in a 37 °C incubator for 30 min to polymerize and form collagen gels. The collagen gels were then aspirated off the PDMS surface, leaving a thin film of random collagen fibrils behind which was then washed gently with water and dried before use. To prepare monomeric collagen coated surfaces, 1 mL of a 50 μg/mL collagen solution was added to the PDMS ring and placed in a 37 °C incubator for 30 min to allow collagen monomer adsorption to the PDMS spin-coated coverslips. Then, the collagen solution was removed by aspiration, washed with water, and dried before use.

### 2.3. Decorin Coating of Aligned Collagen Fibril Micropatterns and Other Collagen Surfaces

To prepare the decorin coating solution, a stock solution of 20 μM bovine articular decorin (D-8428; Sigma, St. Louis, MO, USA) in 1× phosphate-buffered saline (PBS) was diluted to 100, 500, or 1000 nM in 1× PBS for 10 min at room temperature. To coat micropatterns of aligned collagen fibrils with decorin, 500 μL of the decorin solution was withdrawn through the PDMS channel after initial washing with 1× PBS and placed in an incubator for 1 h at 37 °C. Additional 1× PBS was withdrawn for a second wash, after which the PDMS channel was removed, and the fibrils were allowed to dry. To coat collagen monomers or random collagen fibrils with decorin, 500 μL of decorin solution was added into the PDMS ring encircling the collagen. Substrates were placed in an incubator for 1 h at 37 °C to allow the decorin to coat the collagen fibrils, after which each substrate was washed briefly with 1× PBS before drying.

### 2.4. Corneal Cell Culture

Cell experiments were performed with primary rabbit corneal keratocytes (NRK) as previously described [27,28,29]. Briefly, primary NRK were isolated from New Zealand White Rabbit eyeballs (Pel-Freez Biologicals; Rodgers, AR, USA) and cultured in serum free media consisting of Dulbecco’s modified Eagle’s medium (DMEM) supplemented with 100 μM nonessential amino acids (Invitrogen; Gibco, Waltham, MA, USA), 1% penicillin streptomycin (Invitrogen; Gibco), and 1% RPMI vitamin mix for 5 days before seeding onto the collagen substrates. For all experiments, NRK were seeded at 20,000 cells/mL in serum free media and cultured on protein patterns for 5 days. For some substrates, TGF-β1 (T7039; Sigma) was added to the cell culture once at the time of seeding at either 5 ng/mL or 10 ng/mL, 5 days prior to fixation. In some experiments, decorin was added to cell culture media directly following seeding at 2.5 μg/mL (or 25 nM) to compare free decorin in solution to decorin incorporated as a coating to collagen substrates.

### 2.5. Fluorescent Imaging of Collagen and Decorin Coating

To visualize the decorin coating, some experiments utilized decorin conjugated with Alexa Fluor 647. Decorin was labeled with Alexa Fluor 647 via a protein labeling kit according to manufacturer’s protocol (Invitrogen; Carlsbad, CA, USA). For these experiments, aligned collagen fibril micropatterns were coated with a solution of the fluorescent decorin diluted 1:100 in 1× PBS. Substrates were then counterstained for collagen with 5-([4,6-Dichlorotriazin-2-yl]amino) fluorescein hydrochloride (DTAF) and imaged with a Zeiss Axio Observer Z1 inverted microscope (Carl Zeiss, Thornwood, NY, USA) using a 63× oil immersion plan-apochromatic objective (NA = 1.4) and an Orca Flash 4.0 monochrome CMOS camera (Hamamatsu Photonics; Bridgewater, NJ, USA).

### 2.6. Immunofluorescent Staining and Imaging

At the end of each cell culture experiment, corneal cells were fixed with 3% paraformaldehyde for 15 min, permeabilized with 0.05% Triton X-100 for 20 min and blocked with 1 wt% bovine serum albumin (BSA) for at least 1 h at room temperature. Samples were then incubated with primary antibody for α-SMA (A5228; Sigma) in 1 wt% BSA (1:600) for 2 h at 37 °C. Subsequently, samples were incubated with Alexa Fluor 488 conjugated secondary antibodies (A11001; Invitrogen) in 1 wt% BSA (1:200). F-actin was stained with Alexa Fluor 647 conjugated phalloidin (A22287; Invitrogen), and all images were counterstained with DAPI for the nucleus. Fluorescent imaging was performed with a Zeiss Axio Observer Z1 inverted microscope using a 20× plan-apochromatic objective (NA = 0.75) and an Orca Flash 4.0 monochrome CMOS camera.

### 2.7. Cell Morphology Measurements

Morphometric measurements of NRK after 5 days of culture on collagen substrates were acquired using the FIJI distribution of ImageJ (version 1.54f) from fluorescent images of phalloidin actin staining. Images were converted to 8-bit, adjusted for contrast, and converted to binary with a set threshold prior to selection of cells using the “wand (tracing) tool”. Measurements of projected cell area, perimeter, Feret’s diameter and aspect ratio were then taken using the “measure” plugin. The projected area and perimeter of the convex hull area of NRK was then measured by manually drawing straight lines between cell projections. Cell solidity was then calculated as the quotient of cell area divided by convex hull area. Finally, orientation index (*OI*) of individual cells was calculated with the equation

(1) $$OI=2*\cos^{2}\left(\theta -\omega \right)-1,$$

where *θ* is the angle between the maximum major axix and the direction of collagen fibril alignment *ω*. *OI* was used as a measure of cell alignment where 1 ≤ *OI* ≤ −1 with *OI* = 1 indicating 100% alignment, *OI* = 0 indicating no alignment, and *OI* = −1 indicating perpendicular alignment.

### 2.8. Statistics

Statistical analysis was performed using GraphPad Prsim 8 (GraphPad; San Diego, CA, USA). Data are represented as mean ± standard deviation with data points representing the average of measurements from individual experimental replicates. One or two-way ANOVA with Tukey’s post hoc test was used to determine statistical significance between experimental groups with a significance level of *p* < 0.05. A Shapiro–Wilk test was used to assess residual normality.

## 3. Results

### 3.1. Incorporation of Decorin into Aligned Collagen Fibril Patterns

**Figure 1:**
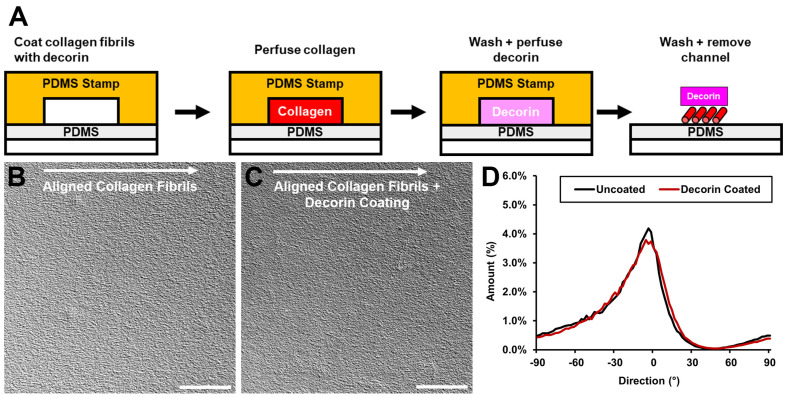
Incorporation of decorin with aligned collagen fibrils. (**A**) Schematic depicting cross-section of microfluidic assembly during key steps of the fabrication of aligned collagen fibrils coated with decorin. Representative differential interference contrast (DIC) microscopic images of (**B**) uncoated and (**C**) decorin-coated aligned collagen fibrils. Arrows depict the direction of flow through the microfluidic channels. (**D**) Distribution of angles in (**B**,**C**) demonstrating most fibrils are aligned around 0 degrees in the direction of shear through the microfluidic channel. Scale bar = 100 μm.

To simulate both the aligned topography and proteoglycan composition of the in vivo corneal stroma ECM, we fabricated micropatterns of aligned collagen fibrils with decorin incorporated after fibril polymerization. Aligned collagen micropatterns were fabricated by perfusing a collagen solution at well-defined shear rates through a microfluidic channel bonded to a PDMS-coated glass cover slip [27,28]. Next, the aligned collagen fibrils were coated with decorin by perfusing a solution of decorin in PBS through the microfluidic channel over the previously deposited aligned fibrillar collagen, incubating for 1 h at 37 °C, and washing with ultrapure water (Figure 1). The microfluidic channel was then removed and the decorin-coated aligned collagen fibril micropatterns were allowed to dry prior to use.

**Figure 2:**
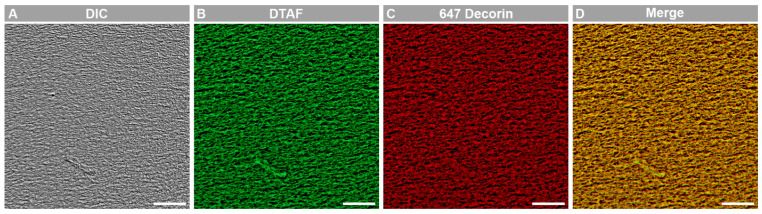
Characterization of decorin coating of aligned collagen fibrils. Representative 63× Oil DIC and fluorescent microscopic images of (**A**) aligned collagen, (**B**) collagen stained with DTAF, (**C**) Alexa Fluor 647 labeled decorin coated on aligned collagen fibrils, and (**D**) a merge of (**C**,**D**). Scale bar = 20 μm.

The alignment of collagen fibril micropatterns was characterized using the ImageJ directionality plugin to analyze differential interference contrast (DIC) images, which demonstrated a similar distribution of angles in both decorin-coated and uncoated micropatterns (Figure 1). To confirm incorporation of decorin via this method, in some experiments decorin was conjugated with Alexa Fluor 647, while the collagen fibril micropatterns were labeled with DTAF [27]. Fluorescent imaging of the resulting micropatterns demonstrated decorin presence on the collagen fibrils (Figure 2). Thus, we confirmed that incorporation of a decorin coating produced micropatterns possessing both aligned topographical cues and the presence of decorin, which we use to investigate TGF-β1 activation of corneal keratocytes.

### 3.2. Decorin Coating Stability and Desorption

To determine the stability of the decorin coating onto the aligned collagen fibrils we performed another set of experiments in which we coated the aligned collagen fibrils with fluorescently labeled decorin. Quantification of the average intensity over time of a coating of Alexa Fluor 647 labeled decorin on aligned collagen patterns showed a roughly 90% retention of fluorescence signal after 48 h and 90% retention after 120 h of incubation in PBS at 37 °C (Figure S1). This result indicates that the decorin coating remains bound to the aligned collagen fibrils during this time frame.

### 3.3. Effect of the Decorin Coating on Keratocyte Differentiation in Response to TGF-β1 Stimulation

**Figure 3:**
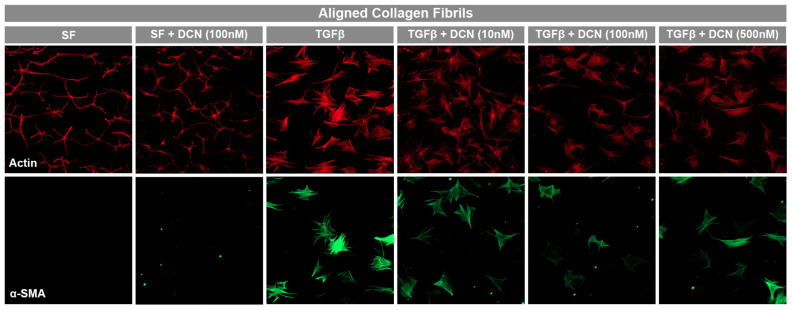
Dependence of keratocyte activation on decorin coating concentration. Representative fluorescent images from 3 independent experiments of corneal keratocytes cultured on uncoated and decorin-coated aligned collagen fibrils in serum free (SF) and TFG-β containing media. Red = actin, green = α-SMA. Substrates were coated with different solutions of decorin (10, 100, or 500 nM). Scale Bar = 100 μm.

**Figure 4:**
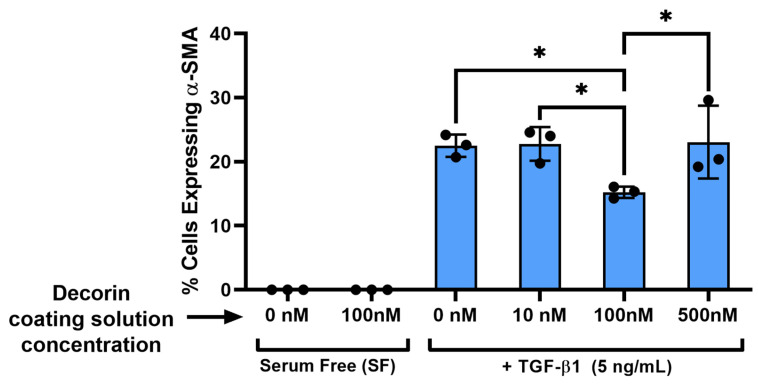
Quantification of α-SMA expression in response to decorin coating concentration. Plot of the mean ± s.d. percentage of cells expressing α-SMA immunofluorescence on the aligned collagen fibrils from 3 experimental replicates. Individual datapoints represent the average percent of cells expressing α-SMA analyzed for each experimental replicate. two-way ANOVA with Tukey’s post hoc test was used to determine significance between experimental groups (* *p* < 0.05).

Transforming growth factor beta (TGF-β1) is a growth factor that is known to mediate the differentiation of quiescent keratocytes into contractile myofibroblasts during wound healing. The response of normal rabbit keratocytes (NRK) to TGF-β1 and the effect of decorin on this response was measured via immunofluorescent staining for α-smooth muscle actin (α-SMA), which has been used extensively as a marker for myofibroblast differentiation [30]. To optimize the decorin coating for this application, decorin solution concentrations from 0 to 500 nM (0 to 50 μg/mL) were first tested. In serum free media without TGF-β1, NRK appear stellate and spindle-like with no α-SMA expression both with and without the decorin coating (Figure 3). However, in the presence of TGF-β1 (5 ng/mL), NRK exhibit greater spreading, stress fiber development, and stain positive for α-SMA (Figure 3). Following quantitative analysis, expression of α-SMA on decorin-coated aligned collagen fibrils was found to decrease significantly by 32% (*p* < 0.05) of α-SMA positive cells on uncoated aligned collagen fibrils for the fibrils coated with a 100 nM decorin solution (Figure 4). Interestingly, a higher concentration of 500 nM did not decrease α-SMA expression.

### 3.4. Effects of Topography and Fibril Alignment on Decorin Coating Inhibition of TGF-β1 Mediated Activation

**Figure 5:**
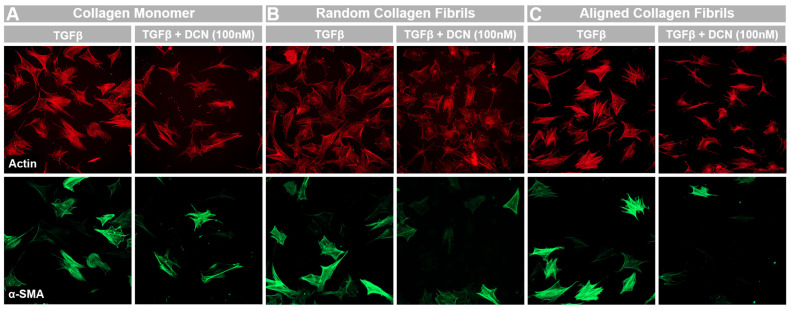
Effects of topography and fibril alignment on decorin coating inhibition of TGF-β1 mediated activation. Representative fluorescent images from 4–5 independent experiments of corneal keratocytes cultured on uncoated and decorin-coated (**A**) collagen monomers, (**B**) random collagen fibrils, and (**C**) aligned collagen fibrils. Red = actin, green = α-SMA Scale Bar = 100 μm.

**Figure 6:**
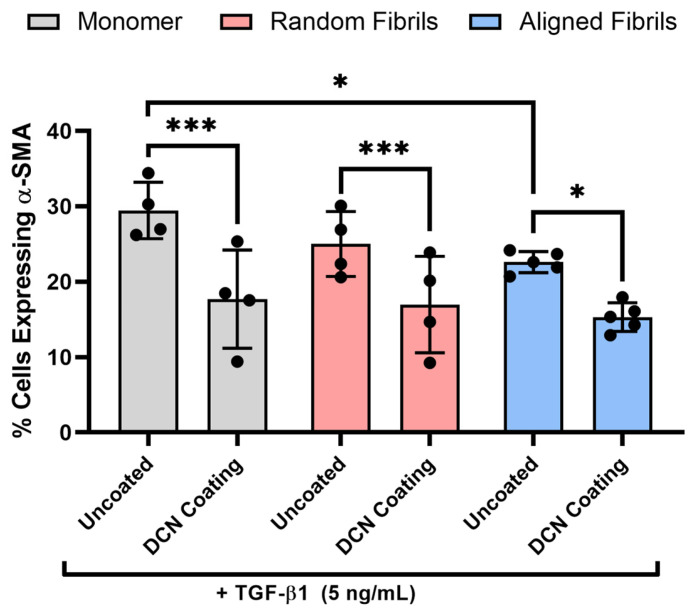
Quantification of α-SMA expression on different collagen topographies coated with decorin. Plot of the mean ± s.d. percentage of cells expressing α-SMA immunofluorescence on the different collagen substrates from 4–5 experimental replicates. Individual datapoints represent the average percent of cells expressing α-SMA analyzed for each experimental replicate. A two-way ANOVA with Tukey’s post hoc test was used to determine significance between experimental groups (* *p* < 0.05, *** *p* < 0.001).

Previous studies investigating the effects of decorin coating on cell behavior have typically been performed on unpolymerized collagen monomers or non-aligned collagen fibrils. To compare the results of the current study with previous findings, α-SMA expression was measured via immunofluorescent imaging in NRKs cultured on collagen monomeric, random fibril, and aligned fibril surfaces for 5 days in the presence of TGF-β1 (Figure 5). No α-SMA expression was observed for negative control experiments when NRK were cultured in serum free media in both uncoated and decorin-coated samples. We observed similar drops in the % of α-SMA positive cells on decorin-coated monomeric (40%), random fibril (32%), or aligned fibril (32%) collagen surfaces (Figure 6), suggesting that decorin can bind to both monomeric and fibrillar collagen and is able to inhibit TGF-β1 mediated myofibroblast activation in both cases. Interestingly, we saw a significant decrease (*p* < 0.05) in the α-SMA expression in the presence of TGF-β1 between uncoated aligned collagen fibrils and uncoated collagen monomers, which suggests that aligned collagen fibril topography on its own has an inhibitory effect on this mediation when compared to collagen monomers. This finding is consistent with previous studies and points to the importance and role of the alignment of collagen in the corneal stroma in regulating cell behavior during the wound healing process [11,31]. This phenomenon emphasizes the need for models accounting for alignment of collagen fibrils to study the role and activity of molecules such as decorin during corneal wound healing.

### 3.5. Decorin Coating Inhibition of TGF-β1 Mediated Activation of Keratocytes Is Partially Independent from TGF-β1 Concentration on Aligned Collagen Fibrils

**Figure 7:**
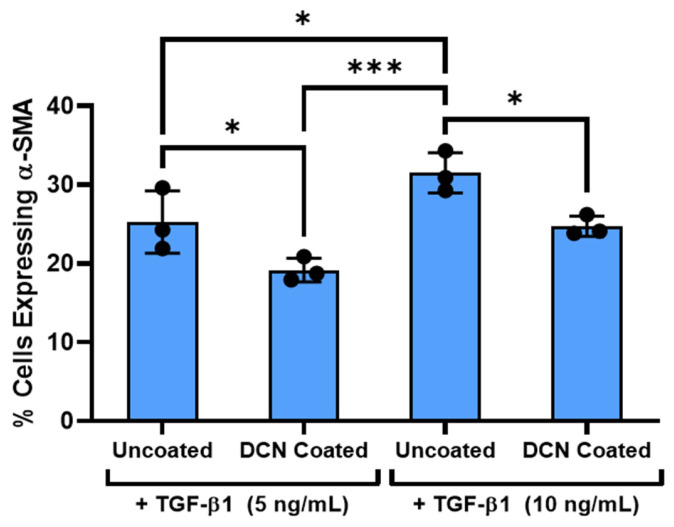
Effect of TGF-β1 concentration on decorin coating inhibition of keratocyte activation. Plot of the mean ± s.d. percentage of cells expressing α-SMA immunofluorescence on the different collagen substrates from 3 experimental replicates. Individual datapoints represent the average percent of cells expressing α-SMA analyzed for each experimental replicate. A two-way ANOVA with Tukey’s post hoc test was used to determine significance between experimental groups (* *p* < 0.05, *** *p* < 0.001).

Previous literature has suggested that decorin inhibits the TGF- β1 signaling pathway through its competitive binding to TGF-β1 in solution which reduces the amount of TGF-β1 available for cell receptor binding [24,32]. Further studies have elaborated on this finding, observing that decorin immobilized on collagen can sequester or inactivate TGF-β1 [33]. Direct interaction of decorin with cell surface receptors (e.g., PDGFR, EGFR, or LRP-1) has also been reported as a pathway for regulation of growth factor signaling by decorin [34,35]. To probe the mechanism of TGF-β1 inactivation by the decorin coating on aligned collagen fibrils, we measured the expression of α-SMA on coated and uncoated aligned collagen fibrils with double the concentration of TGF-β1 (10 ng/mL) than used in the previous experiments (5 ng/mL). By doubling the amount of TGF-β1 in the cell culture media, we expected the extra growth factor to overcome the inhibitory effects of the decorin coating due to the sheer number of TGF-β1 molecules (200 pM and 400 pM for 5 ng/mL and 10 ng/mL, respectively) compared to decorin molecules (37.5 pM estimated decorin coated onto aligned collagen fibrils with a 100 nM solution) [36]. Interestingly, we observed a significant (*p* < 0.05) decrease in the percent of α-SMA expressing cells on decorin-coated collagen fibrils compared to their uncoated counterparts for both the 5 ng/mL (24% drop) and 10 ng/mL (21% drop) concentrations of TGF-β1 (Figure 7). This result suggests that decorin inhibition of α-SMA expression may be in part independent from TGF-β1 concentration and may be influenced by decorin interactions with cell surface receptors. However, additional experiments are required to determine which cell surface receptors decorin maybe interacting with.

### 3.6. Decorin Coating Inhibition of TGF-β1 Mediated Activation of Keratocytes Is Dependent on Immobilization of Decorin to Aligned Collagen Fibrils

**Figure 8:**
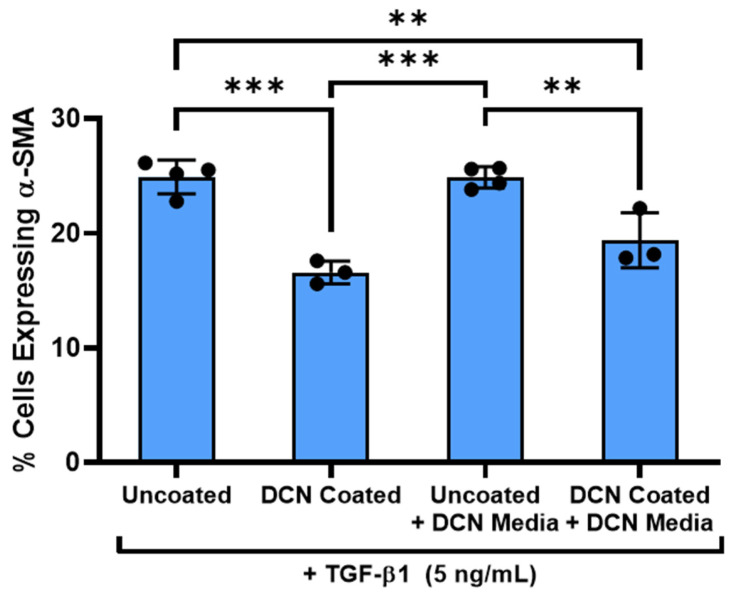
Decorin incorporation with collagen fibrils is necessary for keratocyte activation inhibition. Plot of the mean ± s.d. percentage of cells expressing α-SMA immunofluorescence on the different collagen substrates from 3–4 experimental replicates. Individual datapoints represent the average of cells expressing α-SMA analyzed for each experimental replicate. A one-way ANOVA with Tukey’s post hoc test was used to determine significance between experimental groups (** *p* < 0.01, *** *p* < 0.001).

To further investigate the underlying mechanism for the inhibition of TGF-β1 signaling by the decorin coating, we compared the effects of the same bulk amount (5 μg) of decorin added to the coating solution or added directly to culture media on α-SMA expression by NRK on aligned collagen fibrils. Similar to the observations of Zhang et al. in their study on the inhibition of TGF-β1 induced hypertrophic skin fibroblast contraction by decorin, the decorin in media did not have inhibitory effects (0.14% drop), while the decorin coating demonstrated a significant (*p* < 0.05) reduction in the number of α-SMA expressing cells (33% drop) (Figure 8) [33]. There are several possible explanations for this outcome. First, it may be that decorin immobilized on collagen fibrils can more strongly sequester and prevent the signaling of TGF-β1, while decorin in media binds reversibly with TGF-β1 and does not substantially inhibit signaling. Second, as mentioned previously, decorin incorporated onto the collagen surface may interact with cell surface receptors such as PDGFR, EGFR, or LRP-1 to inhibit the TGF-β1 signaling pathway. Third, the decorin coating may affect the available sites for focal adhesion binding by the NRK which can in turn affect their response to TGF-β1.

## 4. Discussion

Decorin is a multifunctional ECM proteoglycan expressed in the cornea that is known to bind to many different molecules including collagen and growth factors such as TGF-β1 [3,32,37]. Further, decorin has been found to have an inhibitory effect on TGF-β1 signaling and activation of keratocytes and is widely regarded as playing an important regulatory role in corneal wound healing [3,17,25,32,38,39]. This study’s purpose was to investigate this inhibitory effect of decorin within the specific context of aligned collagen fibril topography analogous to the native corneal stroma ECM. While previous studies have uncovered valuable information on the binding interactions between decorin and collagens [36,40,41], decorin and cell surface receptors [35,42], and decorin and growth factors [32,33], the role of collagen fibril alignment and topography in these interactions remains unclear. In tissue where collagen fibrils are aligned, such as in the cornea, tendon, or tumor microenvironment, consideration of this alignment is important as recent studies have demonstrated significant effects of topographical alignment on cell behavior such as migration [43], contractility [44], differentiation [45], proliferation [46], and activation [31].

In the current study we have developed a high-throughput method for coating aligned collagen fibrils with decorin to provide a novel in vitro model incorporating the alignment of collagen I fibrils with decorin to mimic the native corneal stroma. Expanding upon a microfluidic method of fabricating aligned collagen fibrils described previously, the decorin coating was added via incubation within a microfluidic channel on top of the fibril micropattern [27,28]. This method requires only small volumes (<200 μL) of solution to coat the entire micropattern and thus has the advantage of coating many substrates with relatively small amounts of decorin. Fluorescent labeling of decorin confirmed the presence of decorin after coating, and experiments testing the desorption of the fluorescently labeled decorin from the micropatterns determined that the coating remained bound to the collagen with no significant changes for at least 5 days. Notably, this result differs from a previous study by Sylvester and Ratner, who demonstrated decorin coating of immobilized collagen in which the majority of decorin was released into solution within 24 h [36]. Key differences between their study and ours that may account for this difference include: (i) underlying surface of carbonyl diimidazole (CDI) functionalized poly-2-hydroxyethyl methacrylate (pHEMA) vs. PDMS, (ii) collagen type of rat tail vs. bovine skin, (iii) collagen concentration of 300 μg/mL vs. 1.6 mg/mL, (iv) collagen topography of non-oriented collagen vs. aligned collagen fibrils, and (v) coating methodology of overnight incubation at 4 °C vs. microfluidic perfusion and 1 h incubation at 37 °C. To optimize the coating, the effect of a range of decorin coating solutions was tested on TGF-β1 activation of rabbit keratocytes (NRK) on aligned collagen fibrils after 5 days. Although a coating solution concentration of 100 nM decorin resulted in a decrease in α-SMA expression by the NRK, a similar or increased effect was not observed with the higher concentration of 500 nM decorin. Previous studies characterizing the behavior of decorin in solution have reported decorin dimerization at higher concentrations and subsequently, lower binding affinity for collagen and other ligands as dimers as compared to decorin monomers [47,48]. This observation suggests that coating solution concentration may be a critical determining factor for the interactions of decorin with other proteins and role in corneal wound healing. However, further studies are needed to verify and characterize this interaction in detail.

Additional experiments also demonstrated that the 100 nM decorin coating was similarly effective on random collagen fibrils and collagen monomer coated PDMS surfaces with lower α-SMA on all surface types coated with decorin compared to their uncoated counterparts. These data suggest that the decorin coating inhibition of TGF-β1 is not dependent on underlying collagen topography or alignment. However, we also observed lower α-SMA expression between NRK cultured on aligned collagen and collagen monomers without the decorin coating. This observation supports the notion that topography and alignment influence keratocyte activation, which has been previously repeated by our group as well as others [11,31,43]. Although we saw differences in the α-SMA expression on uncoated collagen surfaces of different topographies, we did not find a difference in the α-SMA expression between their decorin-coated counterparts. This observation could suggest that while alignment alone may reduce α-SMA expression, this effect does not synergize with the decorin coating to further reduce myofibroblast differentiation compared to other collagen topographies (i.e., monomeric, random fibril collagen). Another potential explanation is that amount of decorin bound to the collagen monomer, random fibrils, or aligned collagen fibrils differs from each other and accounts for the differences in topographies by binding or sequestering TGF-β1 accordingly. However, further studies on this topic are needed to fully describe the differences between decorin coating activity on aligned collagen fibrils versus collagen monomers or random collagen fibrils. Another difference we observed was a higher variability in α-SMA expression between experimental replicates of decorin-coated collagen monomers and random fibrils compared to the decorin-coated aligned collagen fibrils. Since the collagen monomers and random fibrils cover a wider area and rely solely on static protein adsorption, this increased variability may be due to decreased homogeneity in the collagen coating the PDMS surface, which then results in lower homogeneity in the decorin coating and higher variability in the resulting inhibition of TGF-β1 signaling.

Various mechanisms for the inhibition of TGF-β1 signaling by decorin have been proposed. Some of the mechanisms suggested by previous studies include (i) sequestering and competitive binding by decorin of TGF-β1 to inhibit its activity [3,32] and (ii) interaction between decorin and TGF-β1 related receptor proteins such as LRP-1 [49,50] or PDGFR to influence TGF-β1 signaling [15]. To further investigate this interaction between decorin and TGF-β1 in cell culture, we performed experiments comparing the response of NRK on decorin-coated and uncoated aligned collagen fibrils to 5 ng/mL and 10 ng/mL of TGF-β1 added to the cell culture media. Doubling the concentration of TGF-β1 resulted in increased α-SMA expression, indicating an increase in TGF-β1 mediated myofibroblast differentiation. With the addition of the decorin coating, however, α-SMA expression was not significantly different between TGF-β1 concentrations and was significantly lower than the uncoated counterpart. These data demonstrate that the inhibitory effect of decorin is retained even in the presence of excessive TGF-β1, which suggests that decorin inhibition of TGF-β1 signaling is not entirely dependent on deactivation through competitive binding.

To further explore the mechanism of TGF-β1 signaling inhibition by decorin, we then compared the response of NRK to TGF-β1 in the presence of either the decorin coating or decorin added directly to the cell culture media. Interestingly, no significant decrease in α-SMA expression was observed when decorin was added to the cell culture media unlike the decrease seen in NRK on decorin-coated aligned collagen fibrils. This result suggests that decorin incorporation with an ECM protein such as collagen plays a role in the inhibition of TGF-β1 signaling. This observation is consistent with a previous study by Zhang et al. which reported that inhibition of TGF-β1 enhanced contraction of skin fibroblasts only occurred when the decorin was incorporated into collagen gels and did not have an effect if added directly to the cell culture media [33].

In summary, we have developed a novel method for coating aligned collagen fibrils with decorin, with the potential to be employed to coat the aligned fibrils with other proteoglycans or ECM components such as lumican or hyaluronan in future studies. In this study, the decorin coating decreased TGF-β1 mediated expression of α-SMA by primary keratocytes independent of the underlying collagen, such as collagen monomers, random collagen fibrils, and aligned collagen fibrils patterned onto PDMS. We also observed a previously reported decrease in α-SMA expression on aligned collagen fibrils compared to collagen monomers without the decorin coating. In response to increased TGF-β1 concentration, the decorin coating still decreased expression of α-SMA. Additionally, we found that decorin added directly to the cell culture experiments alongside TGF-β1 did not have the same effect as the decorin coating and did not decrease α-SMA expression levels.

## 5. Conclusions

Decorin has shown promise in previous studies for reducing fibrosis by reducing cell activation and myofibroblast differentiation in response to TGF-β1. Aligned collagen topography has also been shown to influence corneal cell behavior and is a key property of the corneal stroma microenvironment. However, the mechanism by which decorin inhibits TGF-β1, particularly within the context of aligned collagen fibril topography found in the cornea, is not well understood. By fabricating micropatterns of aligned collagen fibrils coated with decorin, we were able to investigate the response of primary rabbit corneal keratocytes on aligned collagen fibril topography in the presence of decorin to TGF-β1. In this study we present a method of fabricating aligned collagen fibrils coated with decorin requiring only a small volume of decorin. In cell culture experiments on the decorin-coated aligned collagen fibrils, we show that the presence of decorin lowers expression of α-SMA in keratocytes stimulated by TGF-β1. We also show that while the decorin coating reduced α-SMA expression irrespective of the different collagen topographies, the non-decorin-coated aligned collagen fibrils also reduced α-SMA expression when compared to non-decorin coated collagen monomers. Finally, we observed that decorin not incorporated with collagen fibrils did not reduce TGF-β1 mediated α-SMA expression as seen with the decorin coating. Taken together, these data show for the first time that decorin coated on aligned collagen fibrils reduces TGF-β1 mediated myofibroblast differentiation and demonstrates the importance of cell-ECM interactions for this inhibition, providing a model relevant to the cornea which can contribute to future studies on decorin as a treatment to prevent corneal fibrosis and blindness.

## Data Availability

The data presented in this study are available on request from the corresponding author.

## Supplementary Materials

The following supporting information can be downloaded at: https://www.mdpi.com/article/10.3390/bioengineering12030259/s1, Figure S1. Characterization of decorin coating stability. Figure S2. Effect of TGF-β concentration on decorin coating reduction in α-SMA expression. Figure S3. Effect of decorin in cell culture media compared to the effect of the decorin coating on α-SMA expression.

## Abbreviations

The following abbreviations are used in this manuscript:

α-SMA	Alpha smooth muscle actin
CMOS	Complementary metal oxide semiconductor
DIC	Differential interference contrast
DTAF	5-([4,6-Dichlorotriazin-2-yl]amino) fluorescein hydrochloride
ECM	Extracellular matrix
EGF	Epidermal growth factor
EGFR	Epidermal growth factor receptor
IGF	Insulin-like growth factor
LRP-1	Low density lipoprotein related receptor one
MEM	Minimum essential media
NRK	Normal rabbit keratocytes
OI	Orientation index
PBS	Phosphate buffered saline
PDGF	Platelet derived growth factor
PDGFR	Platelet derived growth factor receptor
PDMS	Polydimethylsiloxane
TGF-β1	Transforming growth factor beta one
