# Supplementary material for: Effect of Decorin and Aligned Collagen Fibril Topography on TGF-β1 Activation of Corneal Keratocytes
Source: Bioengineering (Basel). 2025 Mar 5;12(3):259. doi: 10.3390/bioengineering12030259 (PMC11939610; doi:10.3390/bioengineering12030259)
Supplement: Supplementary file 1 [file bioengineering-12-00259-s001.zip › bioengineering-3471542-supplementary.pdf]

Supplementary Data

for

Effect of Decorin and Aligned Collagen Fibril Topography on TGF-β1 Activation of Corneal Keratocytes

Nathaniel S. Tjahjono [1], Divya Subramanian [1], Tarik Z. Shihabeddin [1], Hudson D. Hicks [1], Victor D. Varner [1,2], W. Matthew Petroll [2,3] and David W. Schmidtke [1,2]

[1] Department of Bioengineering, University of Texas at Dallas, Richardson, Texas 75080

[2] Department of Biomedical Engineering, University of Texas Southwestern Medical Center, Dallas, Texas 75390

3 Department of Ophthalmology, University of Texas Southwestern Medical Center, Dallas, Texas 75090

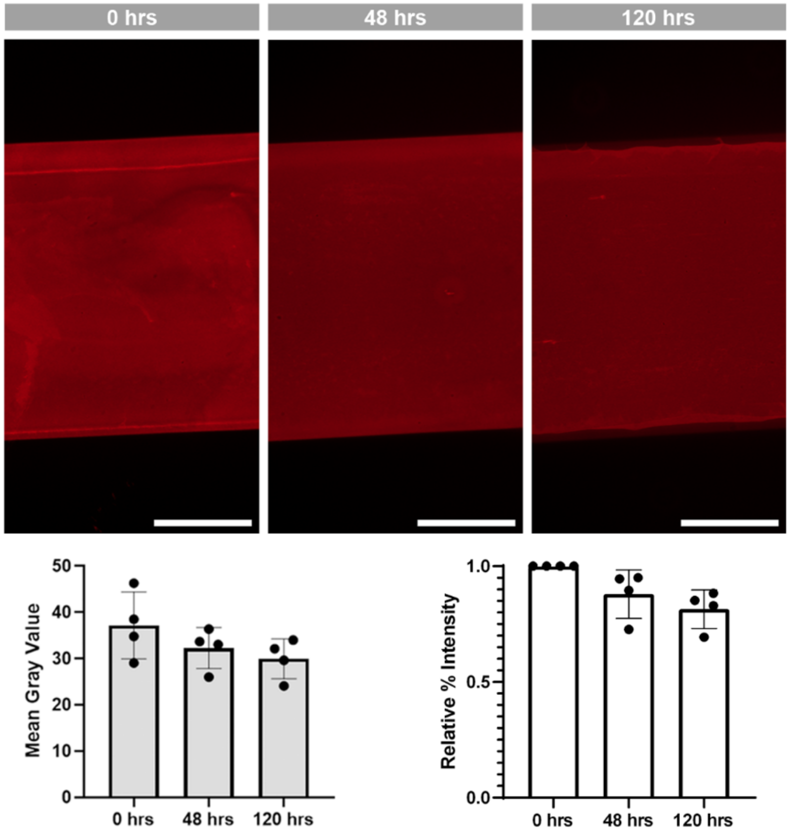

Figure S1. Characterization of decorin coating stability. (A) Representative images of fluorescent Alexa Fluor 647 conjugated decorin coating (red) of aligned collagen fibrils after 0, 48, and 120 hours in 1X PBS. (B) Mean grey values of fluorescent coating image region at each time point from 4 experimental replicates. (C) Relative percent intensity with respect to mean intensity at 0 hours.

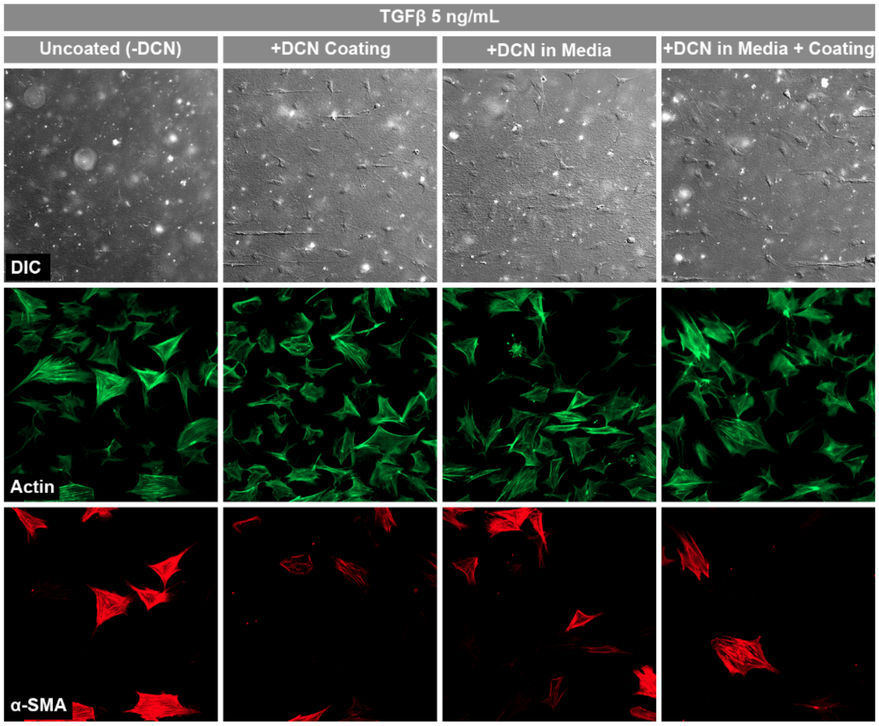

Figure S2. Effect of decorin in cell culture media compared to the effect of the decorin coating on α-SMA expression. Representative DIC and fluorescent images of corneal keratocytes cultured on uncoated and decorin coated aligned collagen fibrils with or without 25 nM decorin added to the cell culture media.

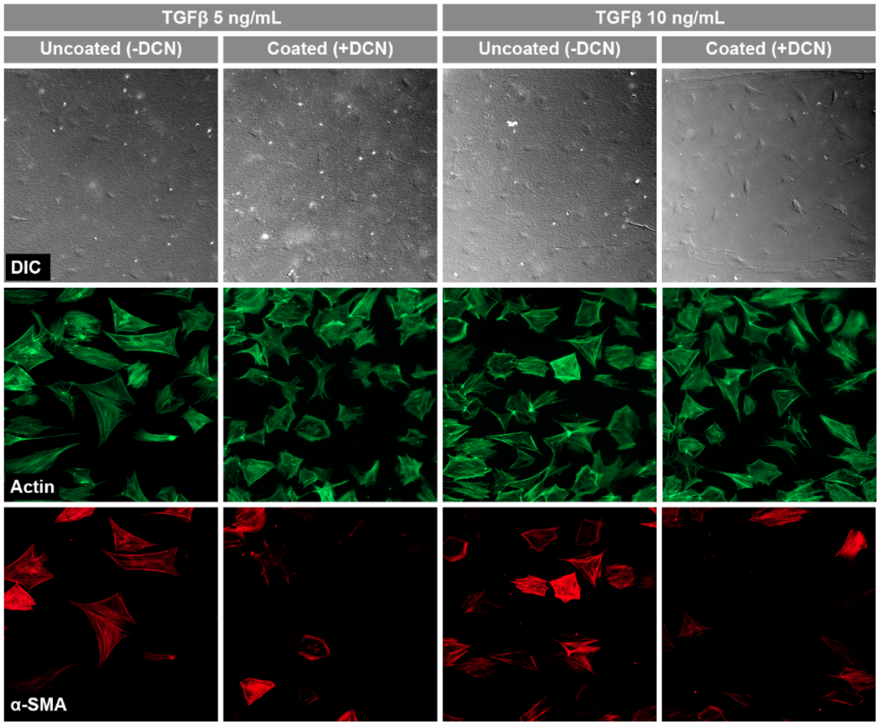

Figure S3. Effect of TGF-β concentration on decorin coating reduction of α-SMA expression. Representative DIC and fluorescent images of corneal keratocytes cultured on uncoated and decorin coated aligned collagen fibrils in the presence of 5 ng/mL of TGF-β or 10 ng/mL of TGF-β in the cell culture media.
